# Topographical Correlation between Structural and Functional Impairment of the Macular Inner Retinal Layers in Multiple Sclerosis Eyes with a History of Optic Neuropathy

**DOI:** 10.3390/jcm12227175

**Published:** 2023-11-19

**Authors:** Vincenzo Parisi, Lucilla Barbano, Giulio Antonelli, Carolina Gabri Nicoletti, Doriana Landi, Giorgia Mataluni, Antonio Di Renzo, Fabio Buttari, Girolama Alessandra Marfia, Diego Centonze, Lucia Ziccardi

**Affiliations:** 1 IRCCS—Fondazione Bietti, Via Livenza 1, 00198 Rome, Italy; vincenzo.parisi@fondazionebietti.it (V.P.); giulio.antonelli@fondazionebietti.it (G.A.); antonio.direnzo@fondazionebietti.it (A.D.R.); lucia.ziccardi@fondazionebietti.it (L.Z.); 2 Multiple Sclerosis Unit, Policlinico Tor Vergata, Viale Oxford 81, 00133 Rome, Italy; carolgabri@gmail.com (C.G.N.); doriana.landi@gmail.com (D.L.); giorgia.mataluni@gmail.com (G.M.); marfia@uniroma2.it (G.A.M.); 3 Department of Systems Medicine, Tor Vergata University, Via Montpellier 1, 00133 Rome, Italy; fabio.buttari@gmail.com (F.B.); centonze@uniroma2.it (D.C.); 4 Unit of Neurology, IRCCS—Neuromed, Via Atinense 18, 86077 Pozzilli, Italy

**Keywords:** multifocal electroretinogram, photopic negative response, retinal ganglion cells, multiple sclerosis, neurodegeneration

## Abstract

We investigated the potential correlation between morphological and functional parameters describing the rarefaction and dysfunction of retinal ganglion cells (RGCs), located in the macula, in multiple sclerosis eyes with a history of optic neuritis (MS-ON). A total of 19 MS-ON eyes from 19 MS patients (mean age: 44.16 ± 4.66 years; 11 females and 8 males), with a mean disease duration of 10.06 ± 6.12 years and full recovery of visual acuity, and 30 age-similar (mean age: 45.09 ± 5.08 years) healthy eyes were submitted for ophthalmological evaluation using swept-source optical coherence tomography (SS-OCT) and multifocal photopic negative response (mfPhNR) to study the structural and functional features of localized RGCs. Both GCL+ thickness (via SS-OCT) and response amplitude density (RAD) (via mfPhNR) measurements were obtained from annular regions and ETDRS sectors. Morphological and electrophysiological data from the control and MS groups were compared by using an ANOVA test. GCL+ values were correlated with the corresponding RADs derived from almost superimposable areas using Pearson’s tests (*p* < 0.01). In MS-ON eyes, the mean values of macular GCL+-T and mfPhNR RAD detected in all rings and ETDRS sectors were significantly reduced (*p* < 0.01) when compared with control ones. In addition, when plotting the GCL+-T and mfPhNR RAD individual data from MS-ON eyes, we found statistically significant linear correlations (*p* < 0.01) when considering responses from both rings and sectors. In conclusion, in MS-ON eyes, a topographical correlation between structural and functional impairment of macular RGCs occurs.

## 1. Introduction

The neurodegenerative disease of multiple sclerosis (MS) is mainly characterized by a chronic demyelination of the central nervous system and can result in visual system involvement, secondary to axonal anterograde injury and retrograde degeneration [1].

In MS patients, after an episode of retrobulbar optic neuritis (ON), rarefaction and functional abnormality of retinal ganglion cells (RGCs) and their axons, considered as the hallmark of retinal morpho-functional degeneration, may occur [2,3,4].

On the structural side, it is possible to identify the thickness reduction of RGCs by acquiring swept-source optical coherence tomography (SS-OCT) scans and by applying the segmentation analysis of the macula, that is the unmyelinated extension of the anterior visual pathways [3]. In fact, due to the ability of this technique to investigate this specific region, over the years, the macula has been selected for understanding mechanisms of neurodegeneration in MS. In particular, the macular ganglion cells complex (GCC), obtained by the combination of retinal nerve fiber layer (RNFL), RGCs, and inner plexiform layer (IPL) thickness, is considered as an accurate and repeatable parameter for the identification of neurodegenerative effects on the macular structure in MS eyes [3,5,6].

On the functional side, RGCs and their axons, which form the innermost retinal layers of the macular area, have been extensively and objectively studied in MS in past years by recording the second harmonic of the focal electroretinogram (FERG) [7] and the pattern ERG (PERG). The PERG amplitude originates from RGCs and fibers and has been recognized as the main electrophysiological parameter able to investigate on the inner retina element function from the whole macular area in different neuro-ophthalmologic disorders [8,9,10,11,12,13,14].

More recently, by exploiting the multifocal paradigm, selective techniques have been developed for studying localized areas of the macular inner retina, such as multifocal pattern ERG (mfPERG) [15] and multifocal photopic negative response (mfPhNR) [16]. Both of these techniques have been introduced to assess the topographical changes of the macular ganglionic elements (RGCs and their axons) with different pattern stimuli. For instance, in MS eyes, a foveal axonal dysfunction has been found, whereas a parafoveal dysfunction has been only described in MS-ON eyes with mfPERG [17]. In contrast, for the first time, we [18] found reduced mfPhNR signals in MS-ON eyes in three different topographical configurations [concentric annular rings centered around the fovea, retinal sectors, and localized areas following the Early Treatment of Diabetic Retinopathy Study (ETDRS)], confirming that inner retinal neurodegeneration in MS-ON eyes involves all analyzed regions (foveal and parafoveal) of the macula, likely resembling a wide phenomenon. 

In the present study, we aimed to investigate the morphology (via SS-OCT) and function (via mfPhNR) of the inner retinal elements from almost superimposable areas of the macula (five concentric rings centered around the fovea and ETDRS sectors) to verify whether there is a topographical correlation between morphological and functional RGC changes in MS-ON eyes.

## 2. Materials and Methods

### 2.1. Study Design and Participants

In this observational retrospective study, all research procedures described in this work adhered to the tenets of the Declaration of Helsinki. The study protocol (N.125/21/FB) was approved by the local ethical committee (Comitato Etico Centrale IRCCS Lazio, Sezione IFO/Fondazione Bietti, Rome, Italy), and upon recruitment, informed consent after full explanation of the procedure was obtained from each subject enrolled in the study.

On one hundred and two patients a diagnosis of relapsing-remitting MS patient was made at the Multiple Sclerosis center of the Tor Vergata University Hospital in Rome.

The demographic and neurological inclusion criteria for enrollment in the study were:

– Age between 30 and 55 years;
– Diagnosis of relapsing remitting MS according to the validated 2010 McDonald criteria [19];
– MS disease duration estimated as the number of years from onset to the most recent assessment of disability, ranging from 5 to 15 years;
– Expanded Disability Status Scale, presenting a ten-point disease severity derived from nine ratings for individual neurological domains [20], ranging from zero to three; this score was assessed by two trained [21] neurologists (D.L. and G.M.);
– Treatment with disease-modifying therapies currently approved for preventing MS relapses, including interferon-β-1a, interferon-β-1b, peginterferon beta-1a, glatiramer acetate, natalizumab, dimethyl fumarate, and teriflunomide [22];
– A single episode of ON treated exclusively with steroid regimen following the recommendations of the Optic Neuritis Treatment Trial [23];
– At least 12 months (ranging from 13 to 20 months) of time elapsed between the onset of ON and inclusion in the study. This criterion was chosen as it is known that retrograde degeneration following ON occurs over a period of 6 months [24].

All 102 MS patients were referred for an ophthalmological evaluation and macular morpho-functional assessment (see below) at the Visual Neurophysiology and Neurophthalmology Research Unit of IRCCS—Fondazione Bietti, between September 2022 and March 2023. 

The ophthalmological assessment was performed in a period less than 2 months after the evaluation at the Multiple Sclerosis Center of Tor Vergata University Hospital in Rome. 

The 102 referred patients were analyzed on the basis of the following inclusion and exclusion ophthalmological criteria.

The inclusion criteria were:

– Mean refractive error (when present) between −3.00 and +3.00 spherical equivalent;
– Intraocular pressure less than 18 mmHg;
– High-contrast BCVA of 0.0 LogMAR for the ETDRS charts.

The exclusion criteria were:

– Presence of central scotoma or square-wave jerks, saccadic intrusions, and nystagmus in the primary position of gaze that can influence the ability to maintain a stable fixation during the mfPhNR recordings (see below);
– Presence of other systemic diseases (i.e., diabetes, systemic hypertension, rheumatologic disorders) that may influence retinal function;
– Presence of glaucoma or other diseases involving the cornea, lens (lens opacity classification system, LOCS III, stage < 1), uvea, or retina.

When an MS patient was affected by ON in both eyes, we studied the longer-affected eye that met the inclusion criteria.

The control group included 30 age-matched healthy subjects (mean age: 45.09 ± 5.08 years, 18 females and 12 males), providing 30 normal eyes, as selected from the outpatient clinic of our ophthalmological department. 

Also, as for controls, the inclusion ophthalmological criteria were:

– Mean refractive error (when present) between −3.00 and +3.00 spherical equivalent;
– Intraocular pressure less than 18 mmHg;
– High-contrast BCVA of 0.0 LogMAR for the ETDRS charts;

The exclusion criteria were:

– Presence of central scotoma or square-wave jerks, saccadic intrusions, and nystagmus in the primary position of gaze that can influence the ability to maintain a stable fixation during the mfPhNR recordings (see below);
– Presence of other systemic diseases (i.e., diabetes, systemic hypertension, rheumatologic disorders) that may influence the retinal function;
– Presence of glaucoma or other diseases involving the cornea, lens (lens opacity classification system, LOCS III, stage < 1), uvea, or retina.

Based on the inclusion/exclusion criteria, the macular morphological (by Optical Coherence Tomography) and functional (by mfPhRN) assessments were performed in each enrolled MS-ON patient (see point 3) and in each control on the same day. 

### 2.2. Optical Coherence Tomography 

**Figure 1:**
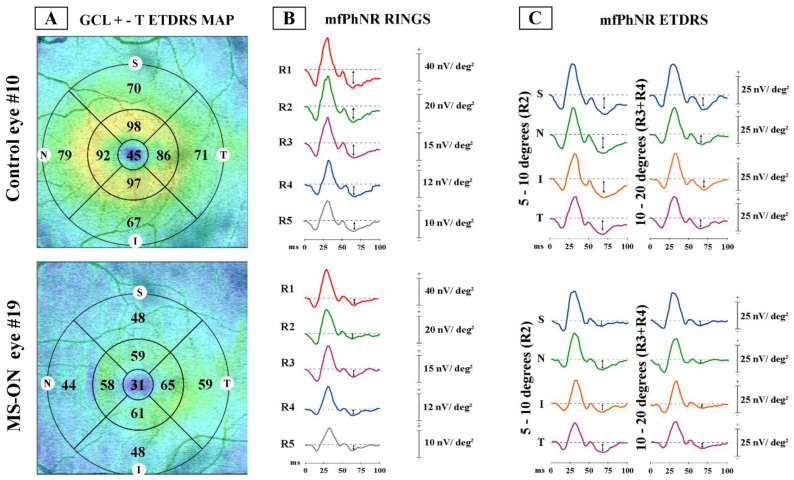
Examples of ganglion cell layer+ thickness (GCL+-T) and multifocal photopic negative response (mfPhNR) data observed in one representative control eye (#10) and in one multiple sclerosis with a history of optic neuritis eye (#19).

The morphological evaluation of inner retinas was conducted by performing a swept-source optical coherence tomography (SS-OCT) automatic segmentation of GCL+, including the retinal nerve fibers layer (RNFL), ganglion cells layer (GLC), inner plexiform layer (IPL), and inner nuclear layer (INL) using a Topcon DRI OCT Triton device (Topcon, Japan). SS-OCT scans were obtained in a dark room after pupil dilation with 1% tropicamide eye drops, and each scan was carefully reviewed for the accurate identification and segmentation of the retinal layers by two expert graders (L.B., L.Z.) to exclude cases of failed segmentation. Quality control and APOSTEL recommendations according to the published criteria were followed [25]. The OCT image quality signal strength index of the acquired scan was at least 50. Scans that did not fulfil the above criteria were excluded from the analysis. A macula 3D scan (V) protocol was used for the segmentation of the macular inner retina layers. The automatic segmentation of averaged GCL+ thickness (GCL+-T) measurements was obtained from two topographical regions (see Figure 1):

(1) Ring analysis: the 1 mm central area (named as Area 1), the parafoveal 1–3 mm ring (named as Area 2, as a mean value of 4 averaged sectors), and the perifoveal 3–6 mm ring (named as Area 3, as a mean value of 4 averaged sectors);
(2) ETDRS Sectors analysis: a 3D model of the retina was computed automatically, and the GCL+ thickness (GCL+-T) was assessed for each of the subfields (within 1, 3, and 6 mm, respectively) as defined by the Early Treatment Diabetic Retinopathy Study (ETDRS). The mean values of GCL+ from ETDRS regions between 1 and 3 mm (Area 2) and between 3 and 6 mm (Area 3) were calculated, averaging the superior (sup), nasal (nas), inferior (inf), and temporal (temp) values separately.

### 2.3. Multifocal Photopic Negative Responses Recordings

The mfPhNR data were recorded by using a modified version of an Espion system (Diagnosys UK, Ltd.; Histon, Cambridge, UK), as reported in our previous work [18].

The multifocal stimulus consists of a circular stimulus of 60 elongated scaled dart pattern “segments” presented in a monitor screen (size, 69 cm width and 38 cm height), with a mean background luminance of 200 cd/m^2^, at the viewing distance of 33 cm. The stimulus frequency was 7 Hz. A video showing the visual stimuli for the mfPhNR recordings is reported in Supplementary Materials (Video S1).

Each “segment” was independently alternated between black (0 cd/m^2^) and white (400 cd/m^2^) according to an m-sequence of 12 bits. The total recording time was an average of 20 min consisting of several periods of about 30 s each. Between recording periods, the subject was allowed to rest for a few seconds.

The monitor screen presented a central fixation cross that was used as a target, and each patient positively reported that they could clearly perceive the cross-fixation target. The eye’s position was monitored by a video system in the screen of the computer. In all subjects, mfPhNR was binocularly recorded in the presence of pupils that were maximally pharmacologically dilated with 1% tropicamide to a diameter of 7 to 8 mm. Pupil diameter was measured by an observer (L.B.) by means of a ruler and a magnifying lens and stored for each tested eye. The cornea was anesthetized with 0.4% benoxinate eye drops.

MfPhNR was recorded between an active Dawson Trick Litzkow bipolar contact electrode and a reference electrode (Ag/AgCl skin electrode placed on the corresponding outer canthi). A small Ag/AgCl skin ground electrode was placed in the center of the forehead. Interelectrode resistance was lower than 3 KOhms. The signal was filtered (band pass 3–100 Hz) by the Espion system. After automatic rejection of artifacts, the first-order kernel response was considered.

After the whole acquisition, the obtained mfPhNR responses were analyzed. The average response amplitude densities (RADs) of the mfPhNR as expressed in nanoVolt/degree^2^ (nV/deg^2^) were measured as baseline-to-trough, that is, the difference between the pre-stimulus baseline and the more negative point in the trough with an implicit time ranging between 50 and 90 ms from the stimulus onset.

As previously reported [18], the recorded mfPhNR responses were analyzed by using two different topographies centered on the fovea, as follows:

(1) Ring analysis: We used the same analysis proposed in other reports for mfERG responses [26,27,28]. This is composed of five concentric annular areas (rings, R) with increasing eccentricity from the fovea. The first one analyzed is a 5° radius circular area centered on the fovea (Ring 1, R1), the second one analyzed is the annular area enclosed between 5° and 10° (Ring 2, R2), the third one analyzed is the annular area enclosed between 10° and 15° (Ring 3, R3), the fourth one analyzed is the more external annular area between 15° and 20° (ring 4, R4), and the fifth one analyzed the outermost area between 20° and 25° (ring 5, R5);
(2) Sector analysis: Following the SS-OCT analysis of the macula [29], we studied the mfPhNR signals from localized areas corresponding to the ETDRS map configuration [30]. It consists of nine sectors, and the central one analyzes the 5° radius circular area centered on the fovea, corresponding to the R1 of the ring analysis. Other external sectors specifically analyze the superior (sup), nasal (nas), inferior (inf), and temporal (temp) areas within 5° and 10° (R2) from the foveal center. The outermost sectors analyze sup, nas, temp, and inf areas within 10° and 20° (R3 + R4) from the foveal center.

### 2.4. Statistical Analysis

We assumed a Gaussian distribution of our data. The normal distribution was assessed by using the Kolmogorov–Smirnov test.

Sample size estimates were obtained from pilot evaluations performed in 10 eyes from 10 MS-ON eyes and 10 eyes from 10 control subjects other than those included in the current study (unpublished data). The sizing was based on the following mfPhNR R1 RAD values: 26.5 ± 8 nV/deg^2^ for the controls and 14.5 ± 6.7 nV/deg^2^ for MS patients at α = 5% (type 1 error) and power = 80% (β = 20%), providing us with 7 participants for each group. Descriptive statistics are shown as the mean and standard deviation (SD). Morphological and electrophysiological data from controls and MS patients were compared by using ANOVA tests and by considering groups as a factor. 

The control data’s 95% lower limits were used to highlight how many MS eyes showed abnormal GCL+-T and mfPhNR RAD values. The 95% lower confidence limits (CLs) were obtained from the control data. 

Lastly, morphological SS-OCT values were related with the corresponding electrophysiological mfPhNR data in MS patients using Pearson’s tests. A *p*-value of 0.01 was considered statistically significant. SPSS (version 25) and MedCalc V.13.0.4.0 (MedCalc, Mariakerke, Belgium) software were used for the statistical analyses.

## 3. Results

Based on the above-mentioned ophthalmological inclusion/exclusion criteria, of the 102 referred patients, 19 MS patients (mean age 44.16 ± 4.66 years; 11 females and 8 males; mean MS disease duration 10.06 ± 6.12 years, range 5–20 years; mean Expanded Disability Status Scale score 1.54 ± 1.43, range 0–3) with previous histories of ON and with full recovery of high-contrast best-corrected visual acuity (BCVA), providing 19 eyes total, were enrolled in the study. 

Table 1 reports the demographic and clinical data of control and MS-ON eyes.

Table 2 reports the individual morphological (ganglion cell layer+ thickness) and electrophysiological (multifocal photopic negative response amplitude densities) data observed in multiple sclerosis eyes with a history of optic neuritis as detected in corresponding macular areas or sectors.

**Figure 2:**
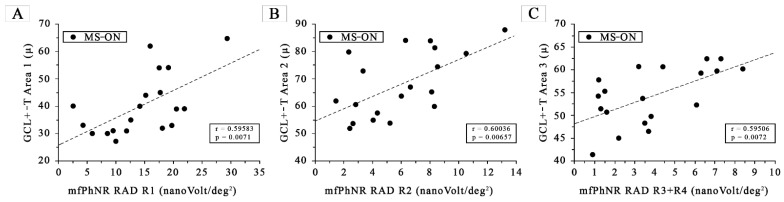
(**A**) The ganglion cells layer complex thickness (GCL+-T) measured in Area 1, plotted as a function of the corresponding values of the multifocal photopic negative responses (mfPhNR) average response amplitude densities (RADs) recorded in Ring 1. (**B**) The ganglion cells layer complex thickness (GCL+-T) measured in Area 2, plotted as a function of the corresponding values of the multifocal photopic negative responses (mfPhNR) average response amplitude densities (RADs) recorded in Ring 2. (**C**) The ganglion cells layer complex thickness (GCL+-T) measured in Area 3, plotted as a function of the corresponding values of multifocal photopic negative responses (mfPhNR) average response amplitude densities (RADs) recorded in Ring 3 + Ring 4.

**Figure 3:**
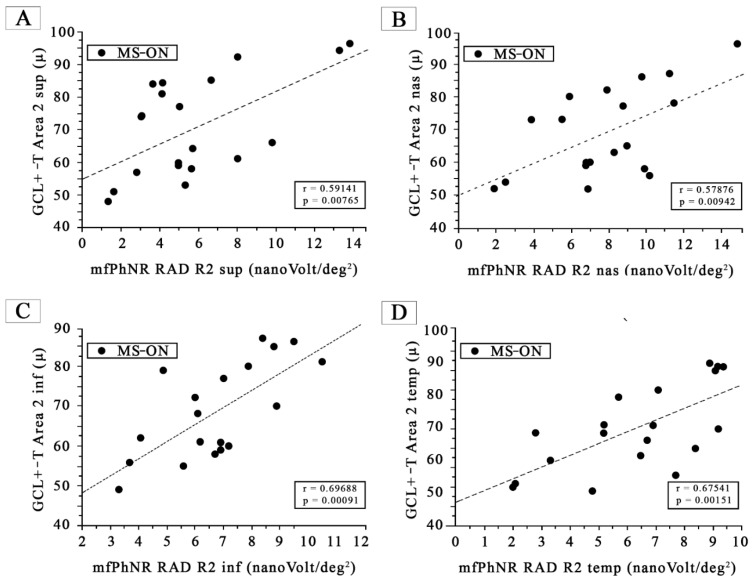
(**A**) The ganglion cells layer complex thickness (GCL+-T) measured in Area 2 superior, plotted as a function of the corresponding values of the multifocal photopic negative responses (mfPhNR) average response amplitude densities (RADs) recorded in Ring 2 superior (R2 sup). (**B**) The ganglion cells layer complex thickness (GCL+-T) measured in Area 2 nasal, plotted as a function of the corresponding values of the multifocal photopic negative responses (mfPhNR) average response amplitude densities (RADs) recorded in Ring 2 superior (R2 nas). (**C**) The ganglion cells layer complex thickness (GCL+-T) measured in Area 2 inferior, plotted as a function of the corresponding values of the multifocal photopic negative responses (mfPhNR) average response amplitude densities (RADs) recorded in Ring 2 inferior (R2 inf). (**D**) The ganglion cells layer complex thickness (GCL+-T) measured in Area 2 temporal, plotted as a function of the corresponding values of the multifocal photopic negative responses (mfPhNR) average response amplitude densities (RADs) recorded in Ring 2 temporal (R2 temp).

**Figure 4:**
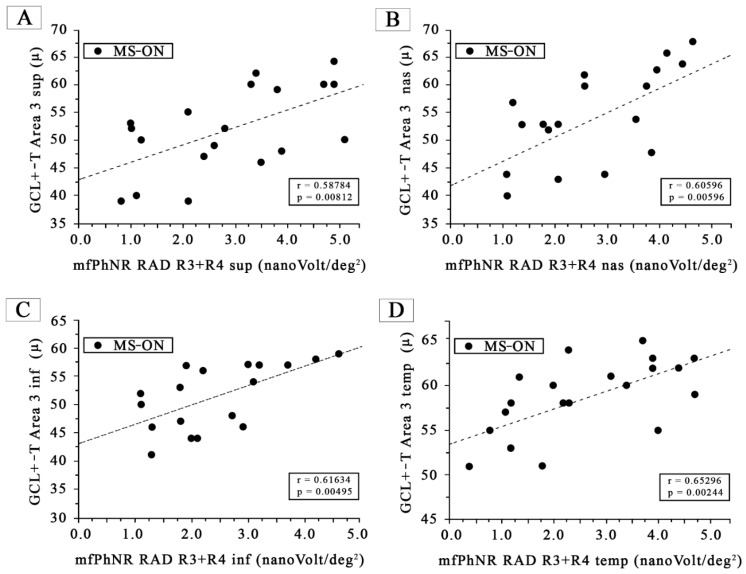
(**A**) The ganglion cells layer complex thickness (GCL+-T) measured in Area 3 superior, plotted as a function of the corresponding values of the multifocal photopic negative responses (mfPhNR) average response amplitude densities (RADs) recorded in Ring 3 + Ring 4 superior (R3 + R4 sup). (**B**) The ganglion cells layer complex thickness (GCL+-T) measured in Area 3 nasal, plotted as a function of the corresponding values of the multifocal photopic negative responses (mfPhNR) average response amplitude densities (RADs) recorded in Ring 3 + Ring 4 nasal (R3 + R4 nas). (**C**) The ganglion cells layer complex thickness (GCL+-T) measured in Area 3 temporal, plotted as a function of the corresponding values of the multifocal photopic negative responses (mfPhNR) average response amplitude densities (RADs) recorded in Ring 3 + Ring 4 inferior (R3 + R4 inf). (**D**) The ganglion cells layer complex thickness (GCL+-T) measured in Area 3 inferior plotted as a function of the corresponding values of the multifocal photopic negative responses (mfPhNR) average response amplitude densities (RADs) recorded in Ring 3 + Ring 4 temporal (R3 + R4 temp).

Figure 1 shows a representative example of the morphological analysis of GCL+ thickness as assessed by SS-OCT and the corresponding functional analysis of RGCs as assessed by the mfPhNR of localized retinal areas (rings and ETDRS map sectors) in one control eye (#10) and in one eye with MS-ON (#19).

(A) GCL+-T data obtained in the ETDRS map configuration: The central area corresponds to Area 1 (1 mm from the fovea), the annular parafoveal area corresponds to Area 2 (from 1 mm to 3 mm from the fovea), and the annular perifoveal area corresponds to Area 3 (from 3 mm to 6 mm from the fovea). In Area 1 it is reported the central GCL+-T value (micron), in Area 2 and Area 3 are reported 4 values of 4 different sectors (superior, S; nasal, N; inferior, I and temporal, T). By calculating the average of the sectors values, the CGL+-T measure of the entire annular Area 2 and Area 3 is obtained, which is reported in Table 2 for MS-ON eye #19;
(B) MfPhNR response amplitude density [measured in nanoV/degree^2^ (nV/deg^2^) as a baseline to trough with an implicit time ranging between 50 and 90 milliseconds (ms) from the stimulus onset and indicated by an arrow (↕)] ring analysis of averaged traces obtained from five circular areas covering 0–25° from the foveal center. In red are depicted the traces derived from a 5° radius circular area centered on the fovea [Ring 1 (R1)]; in green from an annular area enclosed between 5° and 10° centered on the fovea [Ring 2 (R2)]; in purple from an annular area enclosed between 10° and 15° centered on the fovea [Ring 3 (R3)]; in blue from an annular area enclosed between 15° and 20° centered on the fovea [Ring 4 (R4)]; and in grey from an annular area enclosed between 20° and 25° centered on the fovea [Ring 5 (R5)]. In MS-ON eyes, the mfPhNR RAD of R1 was correlated with the GCL+-T of Area 1 (see Figure 2A), the mfPhNR RAD of R2 was correlated with the GCL+-T of Area 2 (see Figure 2B), and the averaged mfPhNR RAD of R3 + R4 was correlated with the GCL+-T of Area 3 (see Figure 2C);
(C) MfPhNR response amplitude density, measured in nanoV/degree^2^ (nV/deg^2^), averaged traces following the Early Treatment Diabetic Retinopathy Study (ETDRS) analysis obtained from four sectors covering 5–10° (R2) and for four sectors covering 10–20° (R3 + R4) from the foveal center. For R2 and R3 + R4, in blue are shown the mfPhNR ETDRS superior (S) sectors; in green are shown the ETDRS nasal (N) sectors; in orange are shown the ETDRS inferior (I) sectors; and in purple are shown the ETDRS temporal (T) sectors. In MS-ON eyes, the mfPhNR RAD of the R2 and R3 + R4 sectors were correlated with the GCL+-T measures from corresponding sectors of Area 2 and Area 3, respectively (see Figure 3 and Figure 4).

Table 3 reports the mean and SD values of SS-OCT measurements from annular areas and sectors (Area 1, Area 2, Area 3, Area 2 sup, Area 2 nas, Area 2 temp, Area 2 inf, Area 3 sup, Area 3 nas, Area 3 temp, Area 3 inf) and the mfPhNR response amplitude density (RAD) measurement from the ring and sector analysis (R1, R2, R3, R4, R5, R2 sup, R2 nas, R2 temp, R2 inf and R3 + R4 sup, R3 + R4 nas, R3 + R4 temp, R3 + R4 inf) of control and MS-ON eyes and the relative statistical analyses between groups. 

The number and relative percentage of abnormal values for morphological (SS-OCT GCL+-T) and electrophysiological (mfPhNR RAD) parameters are reported in Table 3.

### 3.1. RGCs Morphological Data: SS-OCT GCL+-T Ring Analysis

Considering the individual data and 95% lower confidence limit reported in Table 2, the macular GCL+-T was reduced in 15/19 (78.95%) MS-ON eyes in Area 1 and in 19/19 (100%) MS-ON eyes in Area 2 and Area 3.

On average, statistically significant (*p* < 0.01) reduced mean values of macular GCL+-T in Area 1, Area 2, and Area 3 were detected in the MS-ON Group with respect to the control group (see Table 3).

### 3.2. RGCs Morphological Data: SS-OCT GCL+-T ETDRS Sector Analysis

The GCL+-T was measured in four ETDRS sectors (superior, nasal, inferior, and temporal) of the parafoveal annular area (Area 2) and perifoveal annular area (Area 3). 

Considering the individual data and the 95% lower confidence limit reported in Table 2, in Area 2, GCL+-T was reduced in 17/19 (89.47%) MS-ON eyes in the superior sector, in 18 (94.74%) MS-ON eyes in the nasal sector, and in 19/19 (100%) MS-ON eyes in the inferior and temporal sectors. 

In Area 3, GCL+-T was reduced in 17/19 (89.47%) MS-ON eyes in the superior sector, in 18 (94.74%) MS-ON eyes in the nasal sector, and in 19/19 (100%) MS-ON eyes in the inferior and temporal sectors.

On average, statistically significant (*p* < 0.01) reduced mean values of GCL+-T in all ETDRS sectors were detected in the MS-ON Group with respect to the control group (see Table 3). 

### 3.3. RGCs Functional Data: mfPhNR Rings Analysis 

Considering the individual data and the 95% lower confidence limit reported in Table 2 (for R1, R2, and R3 + R4), reduced values of mfPhNR RAD were observed in 19/19 (100%) MS-ON eyes in R5; in 18/19 (94.75%) MS-ON eyes in R1, R3, and R4; in 17/19 (89.47%) MS-ON eyes in R2; and in 18/19 (94.74%) MS-ON eyes in R3 + R4. 

On average, statistically significant (*p* < 0.01) reduced mean values of mfPhNR RAD were detected in the MS-ON group with respect to the control group for all five rings analyzed (see Table 3). 

### 3.4. RGCs Functional Data: mfPhNR ETDRS Sectors Analysis

The mfPhNR RAD was measured in four ETDRS sectors (superior, nasal, inferior, and temporal) of the parafoveal annular area (Ring 2) and perifoveal annular area (Ring 3 + 4). 

Considering the individual data and the 95% lower confidence limit reported in Table 2, in R2, the mfPhNR RADs were reduced in 17/19 (89.47%) MS-ON eyes in the superior sector, in 18/19 (94.74%) MS-ON eyes in the nasal sector, and in 19/19 (100%) MS-ON eyes in the inferior and temporal sectors. 

In R3 + R4, the mfPhNR RADs were reduced in 19/19 (100%) MS-ON eyes in the superior, nasal, inferior, and temporal sectors.

On average, statistically significant (*p* < 0.01) reduced mean values of mfPhNR RAD were detected in the MS-ON Group with respect to the control group for all of the sectors analyzed (see Table 3). 

### 3.5. Morpho-Functional Correlations in MS-ON Eyes for Almost Superimposable Areas

Based on the data reported in Table 2, considering individual values of MS-ON eyes, a concomitant reduction in GCL+ thickness and mfPhNR RAD was detected in Area 2 (GCL+) and in all sectors of R2 (RAD) and in Area 3 (GCL+) and in the inferior and temporal sectors of R3 + R4 (RAD). 

In some individual MS-ON eyes, we observed an absence of concomitant morpho-functional involvement. Indeed, reduced mfPhNR RADs with concomitant normal GCL+ thickness was observed in three MS-ON (#4, #14, #15) eyes in R1, in two MS-ON (#1, #2) eyes in R3 + R4 superior and in one MS-ON (#15) eye in R3 + R4 nasal.

In contrast, reduced GCL+-T with concomitant normal mfPhNR RADs was observed in two MS-ON (#6, #8) eyes in Area 2 and one MS-ON (#15) eye in Area 3. 

When plotting the individual morphological (GCL+-T) and functional (mfPhNR RAD) data observed in MS-ON eyes, we found statistically significant linear correlations in both ring and ETDRS sector analyses. In details, as shown in Figure 2, when considering Area 1, Area 2, and Area 3 GCL+-T values as a function of R1, R2, and R3 + R4 mfPhNR RAD values, respectively, we found statistically significant (*p* < 0.01) linear correlations.

When plotting GCL+-T values from Area 2 sup, Area 2 nas, Area 2 inf, and Area 2 temp as a function of the mfPhNR RAD values from the parafoveal (R2) ETDRS sectors (R2 sup, R2 nas, R2 inf, and R2 temp), respectively, we found all statistically significant (*p* < 0.01) linear correlations, as shown in Figure 3.

Also, Figure 4 shows a statistically significant (*p* < 0.01) linear correlation between GCL+-T values from Area 3 sup, Area 3 nas, Area 3 inf, and Area 3 temp with corresponding mfPhNR RAD values from the perifoveal (R3 + R4) ETDRS sectors (R3 + R4 sup, R3 + R4 nas, R3 + R4 inf, and R3 + R4 temp).

## 4. Discussion

In the present study, we aimed to investigate the morphology (via SS-OCT) and function (via mfPhNR) of the inner retinal elements from almost superimposable areas of the macula (five concentric rings centered on the fovea and ETDRS sectors) to verify whether there is a correlation between morphological and functional RGC changes in MS-ON patients.

### 4.1. Morphological Changes

As already shown in previous studies, the change in macular ganglion cells complex thickness is a morphological sign of neurodegeneration [2,31,32,33]. With the topographical and segmentation analysis of the central retina (macular area) via OCT, the GCL-IPL (our GCL+) measurement has been identified as the most sensitive morphological parameter among the retinal layers, being reduced in MS disease [34,35].

Also, in our MS-ON eyes, we observed a mean GCL+ thickness reduction in all examined annular areas (Area 1, Area 2, Area 3) and in all ETDRS sectors (Area 2 superior, Area 2 nasal, Area 2 inferior, Area 2 temporal) with respect to the control eyes. Our results are in agreement with a previous study [35] that analyzed rings, quadrants, and ETDRS sectors and found a significant thickness reduction in GC-IPL thickness in MS-ON eyes compared with controls and ON fellow eyes. In addition, Hu et al. [35] described in these MS-ON eyes a particular GC-IPL thickness reduction with a horseshoe-like pattern, defined as “U-Zone” with a portion of an elliptic annulus around the fovea, which described the most profound thickness alteration and had the highest discrimination power for MS-ON. 

As already documented in other systemic and ocular neurodegenerative conditions, like in Alzheimer’s disease [36], pituitary adenoma [37], ethambutol-induced ON [38], glaucoma, and Leber’s neuropathy [39,40], specific thickness reduction patterns of inner retinal layers can be found. This may indicate that the macular RGCs are the most vulnerable and that definite topographical characteristic of damage may be observed depending on the mechanism of the neurodegeneration in different diseases.

Because in our cohort of studied MS-ON eyes we have not identified a specific pattern of morphological involvement (in all rings or sectors there is a significant difference between MS-ON and controls), our data are therefore not in agreement with those observed in other pathological conditions [36,37,38,39,40]. This discrepancy observed in our cohort of patients may be due to the limited number of enclosed cases or due to the strict inclusion and exclusion criteria, above all the recovery of visual acuity.

### 4.2. Functional Changes

In the photopic full-field flash ERG (ff-ERG), the PhNR signal is identified as the negative wave following the b-wave whose amplitude is measured from the baseline to the PhNR trough [41]. The ff-PhNR amplitude has been described as reflecting the activity of RGCs [42], and it has been found to be reduced in MS-ON eyes [43], showing a dysfunction of these cells from the whole retina. 

With the advent of the multifocal paradigm, mfPhNR allows for the determination of dysfunction of RGCs [16] from selective areas enclosed into the macular arcades. The recent application of mfPhNR recordings to MS-ON eyes allowed us to analyze the functional activity of localized macular RGCs for the first time by using different topographies, thus confirming that mfPhNR is a specific electrophysiological method that is able to assess the localized damage of RGCs after an inflammatory event as a sign of retrograde degeneration [18]. 

In the present study, we confirmed in a similar cohort of eyes affected by MS-ON a reduction in mfPhNR RAD in all rings and sectors analyzed as a sign of parafoveal and perifoveal dysfunction of RGCs and their axons. 

Taking together the previous [43], recent [18], and present data, all are in agreement, confirming that the RGCs dysfunction after an inflammatory event in MS is a wide phenomenon involving both more central (macular) and peripheral retinal areas.

The reduction in mfPhNR RAD could be ascribed to inflammation [44,45,46] and/or to dysfunction of Muller glia cells or radial astrocytes in the optic nerve [47,48,49] and, in fact, the Muller and amacrine cells have been described to contribute to the PhNR signal origin in animal models of neurodegeneration [49].

### 4.3. Morpho-Functional Correlations

In our study, we found a statistically significant correlation between morphological and functional parameters indicating RGC damage for all examined macular areas (rings and ETDRS sectors). These data indicate that after an episode of ON in MS, the RGCs degeneration is overall evident at the structural and functional level and involves the whole central retinal area, correlating the cell damage with the cell dysfunction. 

Our results agree with Al-Nosairy et al., who investigated on morpho-functional correlation of localized RGCs abnormalities in MS [17] by using different tools (as mf-PERG and CGL and RNFL thickness). In their study, a significant correlation between foveal mf-PERG N2 amplitude and perifoveal GCL thickness as well as a significant correlation between P1-peak time of parafoveal area (Ring 2) with all morphological parameters (RNFL temporal thickness, parafoveal, and perifoveal GCL thickness) were found, suggesting localized and wide morpho-functional RGC and axonal damage in MS patients.

Previous studies [12,50,51] compared the ability of PERG and visual evoked potentials to detect RGCs and axonal dysfunction in MS patients and correlated these functional data to structural RNFL abnormalities, aiming to assess the ability of these tools to detect retinal damage in MS patients. However, these previous studies [12,13,14,15,16,17,18,19,20,21,22,23,24,25,26,27,28,29,30,31,32,33,34,35,36,37,38,39,40,41,42,43,44,45,46,47,48,49,50,51], founding statistically significant correlations between the PERG amplitude and RNFL thickness, only considered the global functional response of RGCs from the central retina (reduced PERG amplitude) and general axonal damage (RNFL thickness reduction).

With the ability of mfPhNR to record the bioelectrical activity derived from localized macular areas, we identified focal dysfunction secondary to RGCs neurodegeneration [18]. By also associating the SS-OCT segmentation analysis, we were able to selectively measure, using the GCL+ parameter, the cell layer most vulnerable to neurodegeneration and to be preferred with respect to the parapapillary RNFL analyzed in previous works for the diagnosis and monitoring of MS [3]. By applying these innovative techniques (SS-OCT RGC-IPL/INL thickness and mfPhNR RAD), we further confirmed the morphological and functional involvement of RGCs as a sign of neurodegeneration induced by MS-ON. 

However, when examining individual correlations between morphological (GCL+-T) and functional (mfPhNR RAD) data, a topographical correspondence was not found in six MS-ON eyes in which a reduction in mfPhNR RAD was associated with normal GCL +-T and in three MS-ON eyes in which the reduction in GCL+-T was associated with normal mfPhNR RAD. Therefore, it is likely that, in few MS-ON eyes, there is a sectorial macular dysfunction that also occurs in absence of morphological changes and vice versa. From this detailed observation, we can infer that all of Area 2 (superior, nasal, inferior, and temporal) and the Area 3 inferior and temporal sectors were the regions mainly affected by the morpho-functional impairment in MS-ON eyes. This may be explained by the fact that, in the macula, the major number of RGCs are located within 4–5 mm (16°) from the foveal center and that there are more RGCs in the nasal retina than in the temporal retina and in the superior than the inferior retina [52]. It is likely that in the para- and perifoveal areas and specifically in the inferior and temporal sectors, where the RGCs are fewer [52], these elements are more morpho-functionally impaired after an episode of ON in MS.

## 5. Conclusions

In conclusion, our study suggests that in MS eyes with an history of ON and full recovery of VA, the damage induced by neurodegeneration widely involves all macular areas.

In MS-ON eyes, a topographical correlation between structural (reduced GCL+-T) and functional (reduced RADs) impairment of macular RGCs occurs, considering both the annular area or retinal sectors. The absence of a specific pattern of impairment, as observed in other pathological conditions [36,37,38,39,40], may be due to the limited number of enclosed cases and the selected inclusion and exclusion criteria.

Further studies will be necessary to confirm this evidence, also considering different stages of MS, the recovery of VA or not, and eventually the type of visual field defect.

## Figures and Tables

**Table 1:** Demographic and clinical characteristics of controls and multiple sclerosis eyes with a history of optic neuritis.

	Controls (N ^a^ = 30)			MS-ON ^b^ (N ^a^ = 19)		
	Range	Mean	SD ^c^	Range	Mean	SD ^c^
Age (years)	30/55	45.09	5.08	30/55	44.16	4.66
Intraocular pressure (mmHg)	12/17	13.3	1.9	12/17	13.9	2.0
BCVA ^d^ (LogMar)	0/0	0	0	0	0	0
Duration of disease (years)	-----------------			5-20	10.06	6.12
EDSS ^e^ score	----------------			0–3	1.54	1.43

^a^ N = Number of eyes; ^b^ MS-ON = multiple sclerosis eyes with a history of optic neuritis; ^c^ SD = 1 standard deviation; ^d^ BCVA = Best-corrected visual acuity; ^e^ EDSS = Expanded Disability Status Scale.

**Table 2:** Individual morphological (ganglion cell layer+ thickness) and electrophysiological (multifocal photopic negative response amplitude densities) data observed in multiple sclerosis eyes with a history of optic neuritis as detected in corresponding macular areas or sectors.

	Rings/Areas						ETDRS ^a^ Area 2								ETDRS ^a^ Area 3							
	R1 ^d^/Area 1		R2 ^d^/Area 2		R3+4 ^d^/Area 3		SUPERIOR		NASAL		TEMPORAL		INFERIOR		SUPERIOR		NASAL		TEMPORAL		INFERIOR	
	PhNR ^b^ R1 ^d^ RAD ^e^ (nV/ deg^2^) ^g^	GCL+ ^c^ T ^f^ (µ) ^h^	PhNR ^b^ R2 ^d^ RAD ^e^ (nV/ deg^2^) ^g^	GCL+ ^c^ T ^f^ (µ) ^h^	PhNR ^b^ R3+R4 ^d^ RAD ^e^ (nV/ deg^2^) ^g^	GCL+ ^c^ T ^f^ (µ) ^h^	PhNR ^b^ R2 ^d^ RAD ^e^ (nV/ deg^2^) ^g^	GCL+ ^c^ T ^f^ (µ) ^h^	PhNR ^b^ R2 ^d^ RAD ^e^ (nV/ deg^2^) ^g^	GCL+ ^c^ T ^f^ (µ) ^h^	PhNR ^b^ R2 ^d^ RAD ^e^ (nV/ deg^2^) ^g^	GCL+ ^c^ T ^f^ (µ) ^h^	PhNR ^b^ R2 ^d^ RAD ^e^ (nV/ deg^2^) ^g^	GCL+ ^c^ T ^f^ (µ) ^h^	PhNR ^b^ R3+R4 ^d^ RAD ^e^ (nV /deg^2^) ^g^	GCL+ ^c^ T ^f^ (µ) ^h^	PhNR ^b^ R3+R4 ^d^ RAD ^e^ (nV/ deg^2^) ^g^	GCL+ ^c^ T ^f^ (µ) ^h^	PhNR ^b^ R3+R4 ^d^ RAD ^e^ (nV/ deg^2^) ^g^	GCL+ ^c^ T ^f^ (µ) ^h^	PhNR ^b^ R3+R4 ^d^ RAD ^e^ (nV/ deg^2^) ^g^	GCL+ ^c^ T ^f^ (µ) ^h^
**#1**	15.2	44	8.3	81.5	3.5	48.24	6.6	85	5.9	80	9.4	81	7.9	80	4.9	*64*	4.2	66	2.3	64	1.9	57
**#2**	17.8	45	2.3	80	4.4	60.75	4.1	84	7.9	82	7.1	75	4.9	79	3.4	*62*	4	63	3.1	61	3	57
#3	20.6	39	4	55	7.3	62.46	2.8	57	5.5	73	8.4	60	5.6	55	5.1	50	3.9	48	4	55	1.8	47
**#4**	16	*62*	8	84	7.1	59.75	8	92	*14.9*	*96*	6.7	62	9.5	86	3.8	59	4.5	64	4.4	62	4.6	59
#5	10	27	1.4	62	1.2	54.25	1.3	48	10.2	56	2.8	64	4.1	62	1	52	1.4	53	1.1	57	1.8	53
**#6**	18.1	32	*13.2*	88	3.4	53.75	9.8	66	6.8	59	6.9	66	6.2	61	1	53	1.9	52	2	60	1.1	52
#7	14.2	40	8.1	65.5	1.6	50.75	*13.3*	*94*	11.5	78	9.1	80	10.5	81	2.8	52	1.2	57	1.2	58	3.1	54
**#8**	*29.4*	*65*	*10.5*	79.25	1.5	55.25	1.6	51	9	65	5.2	64	8.9	70	2.4	47	3.6	54	1.8	51	2.5	51
#9	19.8	33	4.3	57.5	0.9	41.3	4.9	60	2.5	54	3.3	57	6.9	59	2.1	55	1.1	44	0.4	51	1.3	46
#10	5.9	30	5.2	54	2.2	45	5.3	53	6.9	52	7.7	53	6.7	58	3.5	46	2.1	43	3.9	62	1.3	41
#11	22	39	8.5	74.5	6.3	59.25	3	74	8.8	77	2.1	51	7	77	4.7	60	3.8	60	3.4	60	3.2	57
#12	2.6	40	3.3	73	1.2	57.75	5	77	3.9	73	2	50	3.3	49	0.8	39	2.6	60	0.8	55	4.2	58
#13	4.3	33	2.6	53.75	1.3	51.5	5.6	58	1.9	52	4.8	49	3.7	56	1.2	50	1.8	53	1.2	53	1.1	50
**#14**	17.6	*54*	2.4	52	3.2	60.75	3.6	84	9.8	86	9.2	81	8.8	85	4.9	60	2.6	62	3.7	65	2.2	56
**#15**	19.2	*54*	6.3	84.25	*8.4*	60.25	4.1	81	11.3	87	8.9	82	8.4	87	3.3	60	4.7	*68*	3.9	63	3.7	57
#16	11.9	31	6	63.75	6.6	62.44	8	61	7	60	5.2	66	6.1	68	2.1	39	1.1	40	2.2	58	2	44
#17	12.6	35	6.6	67.25	3.7	46.5	5.7	64	6.8	60	5.7	73	6	72	1.1	40	3	44	2.3	58	2.1	44
#18	8.6	30	8.3	60	6.1	52.28	*13.8*	*96*	8.3	63	6.5	58	7.2	60	2.6	49	2.1	53	4.7	63	2.9	46
#19	9.5	31	2.8	60.75	3.8	49.75	4.9	59	9.9	58	9.2	65	6.9	61	3.9	48	3	44	4.7	59	2.7	48
CL ^i^	23.43	45.62	10.46	91.36	7.36	63.69	12.61	93.47	13.96	91.36	15.57	87.44	11.01	92.38	5.27	61.28	4.91	67.10	6.49	65.59	5.07	60.25

^a^ ETDRS = Early Treatment Diabetic Retinopathy Study map configuration; ^b^ mfPhNR = multifocal photopic negative responses; **^c^** GCL+ = ganglion cell layers complex thickness; ^d^ R1, R2, R3, R4 = concentric annular areas (rings) centered on the fovea. R1 refers to the 5° radius circular area, R2 refers to the annular area enclosed between 5° and 10° centered on the fovea, R3 refers to the annular area enclosed between 10° and 15° centered on the fovea, and R4 refers to the annular area enclosed between 15° and 20° centered on the fovea; ^e^ RAD = response amplitude density; ^f^ T = thickness; ^g^ nV/deg^2^ = nanoV/degree^2^; ^h^ µ = micron; ^i^ CL = 95% lower confidence limit derived from controls. Presented in *italics* are the values considered as “normal” according to the 95% lower confidence limit. Presented in **bold** are the multiple sclerosis eyes with a history of optic neuritis, in which an absence of concomitant normal/abnormal mfPhNR RAD and GCL+ thickness values was found (see also point 3.5).

**Table 3:** Descriptive statistics analysis of morphological (ganglion cell layer + thickness) and electrophysiological (multifocal photopic negative response amplitude densities) data from controls and multiple sclerosis eyes with a history of optic neuritis compared with ANOVA tests.

	Controls (N ^a^ = 30)		MS-ON ^b^ (N ^a^ = 19)		Ab ^c^		ANOVA ^d^	
	Mean	SD ^e^	Mean	SD ^e^	N ^a^	%	f (1.48)	*p*
Area 1 GCL+-T ^f^ (µ ^g^)	48.467	8.080	40.211	11.208	15	78.95%	9.42	0.004
Area 2 GCL+-T ^f^ (µ ^g^)	93.408	5.484	68.211	11.905	19	100.00%	101.40	<0.001
Area 3 GCL+-T ^f^ (µ ^g^)	65.375	4.504	54.314	6.312	19	100.00%	51.24	<0.001
ETRS ^h^ Area 2 sup ^i^ GCL+-T ^f^ (µ ^g^)	95.400	5.164	70.737	15.509	17	89.47%	65.17	<0.001
ETRS ^h^ Area 2 nas ^j^ GCL+-T ^f^ (µ ^g^)	93.767	6.426	69.000	13.367	18	94.74%	75.98	<0.001
ETRS ^h^ Area 2 inf ^k^ GCL+-T ^f^ (µ ^g^)	94.767	6.377	68.737	11.906	19	100.00%	99.29	<0.001
ETRS ^h^ Area 2 temp ^l^ GCL+-T ^f^ (µ ^g^)	89.700	6.052	65.105	10.959	19	100.00%	102.58	<0.001
ETRS ^h^ Area 3 sup ^i^ GCL+-T ^f^ (µ ^g^)	63.133	4.939	51.842	7.712	17	89.47%	39.20	<0.001
ETRS ^h^ Area 3 nas ^j^ GCL+-T ^f^ (µ ^g^)	68.833	4.624	54.105	8.582	18	94.74%	60.95	<0.001
ETRS ^h^ Area 3 inf ^k^ GCL+-T ^f^ (µ ^g^)	61.833	4.227	51.421	5.611	19	100.00%	54.64	<0.001
ETRS ^h^ Area 3 temp ^l^ GCL+-T ^f^ (µ ^g^)	67.700	5.633	58.684	4.204	19	100.00%	35.89	<0.001
R1 ^m^ mfPhNR ^n^ RAD ^o^ (nV/deg^2^) ^p^	26.274	7.267	14.489	6.716	18	94.74%	32.40	<0.001
R2 ^q^ mfPhNR ^n^ RAD ^o^ (nV/deg^2^) ^p^	11.404	4.163	5.900	3.180	17	89.47%	24.19	<0.001
R3 ^r^ mfPhNR ^n^ RAD ^o^ (nV/deg^2^) ^p^	8.163	2.346	3.879	2.420	18	94.74%	37.86	<0.001
R4 ^s^ mfPhNR ^n^ RAD ^o^ (nV/deg^2^) ^p^	4.323	1.082	2.584	1.377	18	94.74%	24.29	<0.001
R5 ^t^ mfPhNR ^n^ RAD ^o^ (nV/deg^2^) ^p^	3.728	0.873	1.263	0.893	19	100.00%	91.13	<0.001
ETDRS ^h^ R2^q^ sup ^i^ mfPhNR ^n^ RAD ^o^ (nV/deg^2^) ^p^	14.437	4.885	5.863	3.451	17	89.47%	44.34	<0.001
ETDRS ^h^ R2 ^q^ nas ^j^ mfPhNR ^n^ RAD ^o^ (nV/deg^2^) ^p^	16.653	7.191	7.832	3.194	18	94.74%	25.27	<0.001
ETDRS ^h^ R2 ^q^ inf ^k^ mfPhNR ^n^ RAD ^o^ (nV/deg^2^) ^p^	13.447	6.525	6.768	1.959	19	100.00%	18.71	<0.001
ETDRS ^h^ R2 ^q^ temp ^l^ mfPhNR ^n^ RAD ^o^ (nV/deg^2^) ^p^	17.520	5.210	6.326	2.483	19	100.00%	76.28	<0.001
ETDRS ^h^ R3+R4 ^u^ sup ^i^ mfPhNR ^n^ RAD ^o^ (nV/deg^2^) ^p^	5.983	1.906	2.874	1.454	19	100.00%	36.85	<0.001
ETDRS ^h^ R3+R4 ^u^ nas ^j^ mfPhNR ^n^ RAD ^o^ (nV/deg^2^) ^p^	5.513	1.626	2.768	1.201	19	100.00%	40.14	<0.001
ETDRS ^h^ R3+R4 ^u^ inf ^k^ mfPhNR ^n^ RAD ^o^ (nV/deg^2^) ^p^	5.710	1.705	2.447	1.017	19	100.00%	56.56	<0.001
ETDRS ^h^ R3+R4 ^u^ temp ^l^ mfPhNR ^n^ RAD ^o^ (nV/deg^2^) ^p^	7.287	2.111	2.689	1.393	19	100.00%	70.41	<0.001

^a^ N = number of eyes; ^b^ MS-ON = multiple sclerosis eyes with a history of optic neuritis; ^c^ Ab = abnormal with respect to 95% lower confidence limits; ^d^ ANOVA = one-way analysis of variance. *p*-values < 0.01 were considered statistically significant for group comparisons; ^e^ SD = 1 standard deviation; ^f^ GCL +-T = ganglion cell layers complex thickness; ^g^ µ = micron; ^h^ ETDRS = Early Treatment Diabetic Retinopathy Study map configuration; ^i^ sup = superior sector; ^j^ nas = nasal sector; ^k^ inf = inferior sector; ^l^ temp = temporal sector; ^m^ R1 = concentric annular areas (rings) analyzing the 5° radius circular area centered on the fovea; ^n^ mfPhNR = multifocal photopic negative responses; ^o^ RAD = response amplitude density; ^p^ nV/deg^2^ = nanoV/degree^2^; ^q^ R2 = annular area enclosed between 5° and 10° centered on the fovea; ^r^ R3 = annular area enclosed between 10° and 15° centered on the fovea; ^s^ R4 = annular area enclosed between 15° and 20° centered on the fovea; ^t^ R5 = annular area enclosed between 20° and 25° centered on the fovea; ^u^ R3 + R4 = annular area enclosed between 10° and 20° centered on the fovea.

## Data Availability

Data are contained within the article.

## Supplementary Materials

The following supporting information can be downloaded at: https://www.mdpi.com/article/10.3390/jcm12227175/s1. Video S1: The video shows the visual stimuli for the mfPhNR recordings. Each “segment” was independently alternated be-tween black (0 cd/m^2^) and white (400 cd/m^2^) according to an m-sequence of 12 bits. Total recording time was av-erage 20 min of several periods of about 30 s each. Between recording periods, the subject was allowed to rest for a few seconds. The monitor screen presented a central fixation cross that was used as a target.

## Abbreviations

MS	Multiple sclerosis
ON	Optic neuritis
MS-ON	Multiple sclerosis patients with optic neuritis followed by good recovery of best-corrected visual acuity
RGCs	Retinal ganglion cells
GCC	Ganglion cells complex
SS-OCT	Swept-source optical coherence tomography
RNFL	Retinal nerve fiber layer
FERG	Focal electroretinogram
PERG	Pattern electroretinogram
mfPhNR	Multifocal photopic negative response
mfPERG	Multifocal pattern electroretinogram
BCVA	Best-corrected visual acuity
RAD	Response amplitude density
GCL+-T	Ganglion cells layer thickness
IPL	Inner plexiform layer
INL	Inner nuclear layer
ETDRS	Early Treatment Diabetic Retinopathy Study
SD	One standard deviation of the mean
N	Number of eyes of each group
ANOVA	One-way analysis of variance
R	Ring
sup	Superior sector
nas	Nasal sector
inf	Inferior sector
temp	Temporal sector
